# The prognostic impact of the immune microenvironment in small-cell neuroendocrine carcinoma of the uterine cervix: PD-L1 and immune cell subtypes

**DOI:** 10.1186/s12935-022-02716-6

**Published:** 2022-11-14

**Authors:** Xiaoying Sun, Lili Liu, Ting Wan, Qidan Huang, Jieping Chen, Rongzhen Luo, Jihong Liu

**Affiliations:** 1 State Key Laboratory of Oncology in South China, Collaborative Innovation Center for Cancer Medicine, Cancer Center, Sun Yat-Sen University, No. 651, Dongfeng Road East, Guangzhou, 510060 China; 2 Department of Gynecology, Guangdong Provincial People’s Hospital, Guangzhou, People’s Republic of China 510080 Guangdong; 3 State Key Laboratory of Oncology in South China, Collaborative Innovation Center for Cancer Medicine, Department of Gynecologic Oncology, Cancer Center, Sun Yat-Sen University, No. 651, Dongfeng Road East, Guangzhou, 510060 China; 4 State Key Laboratory of Oncology in South China, Collaborative Innovation Center for Cancer Medicine, Department of Pathology, Cancer Center, Sun Yat-Sen University, No. 651, Dongfeng Road East, Guangzhou, 510060 China

**Keywords:** PD-L1, FOXP3, TILs, Small-cell neuroendocrine carcinoma of the cervix

## Abstract

**Background:**

We investigate the correlation between programmed cell death-ligand 1 (PD-L1) and tumor-associated immune cell (TAIC) density in small-cell neuroendocrine carcinoma of the uterine cervix (SCNEC) and their correlation with clinicopathologic features.

**Methods:**

PD-L1 and mismatch repair protein (MMR) expression in cancer cells and the density of TAIC were evaluated by immunohistochemistry in 89 SCNEC patients. The combined positive score (CPS), tumor proportion score (TPS), and immune cell score (ICS) of PD-L1 were measured, along with their correlation with clinicopathologic features in SCNEC patients using statistical analyses.

**Results:**

CPS of PD-L1 ≥ 1 was seen in 68.5% of patients, positive TPS and ICS of PD-L1 were detected in 59.6% and 33.7% of patients, respectively. PD-L1^CPS^ was higher in tumor-infiltrating immune cells (r = 0.387, *p* = 0.001) and positively correlated with programmed cell death-1 and forkhead box P3 + regulatory T cell (FOXP3 + Treg) infiltration (r = 0.443, *p* < 0.001; r = 0.532, *p* < 0.001). There was no statistical correlation between PD-L1 and MMR status. PD-L1^CPS^ and PD-L1^ICS^ positivity were independent prognostic factors, correlating with a favorable survival (HR (95%CI) = 0.363(0.139–0.950), *p* = 0.039 and HR (95% CI) = 0.199(0.050–0.802), *p* = 0.023, respectively). PD-L1^ICS^ positivity was an independent indicator of recurrence in SCNEC patients and associated with better disease-free survival (HR (95% CI) = 0.124(0.036–0425), *p* = 0.001). TAIC and MMR levels had no statistical impact on survival results.

**Conclusions:**

PD-L1 positivity was seen in over half of SCNEC tumors. It may work synergistically with FOXP3 + Treg and other infiltrating immune cells to support an adaptive immune response. PD-L1 positivity may be a favorable prognostic factor in SCNEC.

**Supplementary Information:**

The online version contains supplementary material available at 10.1186/s12935-022-02716-6.

## Introduction

Small-cell neuroendocrine carcinoma of the cervix (SCNEC) is a rare but lethal cervical malignancy, constituting 0.9–1.5% of invasive cervical cancers and an annual incidence rate of 0.06 per 100,000 women[1, 2]. SCNEC has highly aggressive biological characteristics with high incidence rates of hematogenous (80%) and lymphogenous (45–57%) metastases [3, 4]. Even with advancements in treatment, SCNEC prognosis is poor (including patients diagnosed at early stages of disease) with survival rates for SCNEC patients at all stages ranging between 11–54% [5]. Thus, identifying prognostic factors and novel therapeutic options are strongly warranted for SCNEC patients.

Several studies suggest that tumor-infiltrating immune cells (TILs) with different cell types and densities play a significant role in the prognoses of many tumors, such as lung, breast, and cervical cancer [6–8]. Programmed cell death-1 (PD-1) regulates effector T cell activity by interacting with one of its ligands, programmed cell death protein 1 ligand 1 (PD-L1), which is expressed on various types of tumor cells including cervical cancer [9], and PD-L1 influences the functional maintenance of regulatory T cells (Tregs), a subset of CD4 + cells [10]. Tregs regulated by forkhead box P3 + (FOXP3 +), a forkhead helix transcription factor, are found in various tumors and are considered as a prognostic factor in human cancers [11–13]. Deficient mismatch repair (dMMR) and PD-L1 positivity have been shown to predict the benefit of anti-PD-1/PD-L1 immunotherapy in several cancers [14–16]. Recently, a case study found that PD-L1 expression, mainly focal, occurred in 70% of SCNEC tumors, and loss of MMR occurred in 33% of SCNEC tumors [17].

However, few studies have explored the expression, infiltration, relationship, and prognostic role of immune and anti-immune factors in SCNEC. In this study, we evaluated and explored the correlation between PD-L1 and tumor-associated immune cell (TAIC) levels in SCNEC tumors and investigated their relationship with SCNEC clinicopathological features and prognosis.

## Materials and methods

### Patients

This study enrolled 89 SCNEC patients who had undergone radical hysterectomy at the Sun Yat-sen University Cancer Center from 2005 to 2019. The last follow-up was in January 2020. The patients were followed up for 1.0–125.7 months. Clinical characteristics were collected including age, the International Federation of Gynecology and Obstetrics (FIGO) stage, tumor size, cervical human papillomavirus (HPV) DNA level, neuron-specific enolase (NSE) serum level, operative reports, histopathology, adjuvant therapy, tumor recurrence, and current status. Staging is based on the FIGO 2018 cervical cancer staging system. This trial was approved by the Sun Yat-sen University Cancer Center Institutional Review Board (YB2019-182–01), where all samples were handled in line with ethical and legal standards.

### Tissue microarray (TMA) construction

TMAs were constructed from SCNEC patient samples that were embedded in paraffin, where representative tumor tissue areas were confirmed by two gynecologic pathologists based on hematoxylin–eosin (H&E) staining. Triplicate cores (1 mm^2^) were randomly punched in the marked tumor areas and placed into recipient paraffin blocks using a tissue microarrayer (Beecher instruments). Then, paraffin blocks were incubated at 37℃ for 15 min (so sample tissues attached to the wax) and cut into 4 mm-thick sections.

### Immunohistochemistry (IHC)

All of the IHC markers were executed on the SCNEC tissue microarray slides following the manufacturer’s instructions (as described in our previous publication [18]). We assessed the expression of the following markers in SCNEC tumor samples: PD-L1 (clone SP263, Ventana, 1:150 dilution), PD-1 (clone NAT105, Abcam, 1:100 dilution), FOXP3 (clone 236A/E7, Abcam, 1:300 dilution), PMS1 homolog 2 (PMS2, clone EP51, Agilent, 1:100 dilution), mutS homolog 2 (MSH2, clone FE11, Agilent, 1:150 dilution), mutL homolog 1 (MLH1,clone ES05, Agilent, 1:50 dilution), mutS homolog 6 (MSH6, clone EP49, Agilent, 1:150 dilution), CD3 molecule (CD3, clone 2GV3, Ventana, 1:150 dilution), membrane spanning 4-domains A1(CD20, clone L26, Ventana, 1:100 dilution), CD4 molecule (CD4, clone SP35, Abcam, 1:50 dilution), CD8a molecule (CD8, clone 144B, Dako, 1:100 dilution), CD68 molecule (CD68, clone MRQ-42, Ventana, 1:100 dilution), marker of proliferation Ki-67 (Ki67, clone MIB-1, DAKO, 1:100 dilution), cyclin dependent kinase inhibitor 2A (P16, clone CINecP16,Ventana, 1:100 dilution).

Immunostaining was reviewed and evaluated by two independent pathologists who were blinded to the patients. The percentage of PD-L1 membrane staining in tumor and immune cells and combined positive score (CPS) were evaluated (minimum of 100 cells were evaluated) [19]. The tumor proportion score (TPS) was defined as the percentage of PD-L1-positive tumor cells. When TPS was ≥ 1% (the median value), tumors were considered as “PD-L1^TPS^ positive”. The immune cell score (ICS) was defined by the percentage of PD-L1-positive immune cells. When ICS was ≥ 5% (the median value), tumors were considered as “PD-L1^ICS^ positive”. The CPS was defined as the total number of all PD-L1-positive cells (including tumor cells, lymphocytes, and macrophages) divided by the number of viable tumor cells and multiplied by 100. When CPS was ≥ 1 (the median value), tumors were considered as “PD-L1^CPS^ positive” [20, 21].

PD-1 staining in immune cells was assessed as present (positive) or absent (negative). And the percentage of CD3, CD20, CD4, CD8, CD68 and FOXP3 positive immune cells was obtained by dividing the total number of CD3, CD20, CD4, CD8, CD68 or FOXP3 positive immune cells by the total number of immune cells present in the tissue section. If the infiltration by percent of immune cells was less than the median value, the tumors were classed as having low immune cell infiltration. In addition, P16 was interpreted as positive if diffuse and block-like staining was found in all cores and negative if there was no or patchy staining in the cores. The loss of MMR proteins was defined as complete absence of nuclear staining. Ki67 index was defined by the percentage of positive-stained tumor cells out of a total number of tumor cells (at least 200 cells were evaluated), where “low proliferation” refers to samples that had positive-stained tumor cell percentages that were below the median value.

### Statistical analyses

The Statistical Product and Service Solutions (SPSS) 25.0 statistical software package was used to perform all statistical analyses. The association between two variables was assessed by the chi-square test and Fisher’s exact test. Spearman’s rank correlation coefficients were used to calculate the bivariate correlations between the studied variables. Survival curves were plotted using the Kaplan–Meier method, and the log-rank test was used to determine the statistical differences. Multivariate analysis was analyzed using the Cox proportional hazards regression model on all significant characteristics determined using univariate analysis. Values were considered statistically significant when *p* < 0.05.

## Results

### Clinical characteristics

The clinicopathological information of the 89 SCNEC patients included in this study is summarized in Table 1. The median age was 44.5 years and the percentages of patients with tumor stages between I–II and III–IV were 59.6% and 40.4%, respectively. The tumor size range was 0.5–9 cm, where almost half of the tumors were of a size that was greater than 4 cm. There were 33 patients (37.1%) receiving neoadjuvant chemotherapy (NACT). Adjuvant chemotherapy was given in 84 (94.4%) patients, which included 55 patients who had also received adjuvant radiotherapy. The cisplatin and etoposide (EP) chemotherapy regimen was the therapy that was used the most in the patients. The total mortality rate was 33.7%, which involved much lower levels in early-stage cancer patients (26.4%) than in advanced-stage cancer patients (44.4%). The rate of tumor recurrence was 46.1%, where patients in early and advanced stages had similar levels.

**Table 1:** Correlation Between PD-L1 and the Clinicopathological Features of SCNEC

Variable	Total cases		PD-L1^TPS*^		*P*-value (r*)	PD-L1^ICS*^		*P*-value (r)	PD-L1^CPS*^		*P*-value (r*)
			Positive	Negative		Positive	Negative		Positive	Negative	
Age (years)	< 45	45	26	19	0.830	16	29	0.823	32	13	0.652
	≥ 45	44	27	17	(0.037)	14	30	(− 0.04)	29	15	(− 0.056)
FIGO* stage	I-II	53	33	20	0.660	18	35	1.000	35	18	0.644
	III-IV	36	20	16	(− 0.067)	12	24	(− 0.007)	26	10	(0.065)
Tumor size(cm)	< 2	11	7	4	0.923 (− 0.043)	4	7	0.932	7	4	0.881
	≥ 2, < 4	38	23	15		12	26	(0.008)	27	11	(-0.003)
	≥ 4	40	23	17		14	26		27	13	
NSE(U/ml)	≤ 15.2	55	31	24	0.804	13	42	0.179	37	18	1.000
	> 15.2	23	14	9	(0.042)	9	14	(0.157)	16	7	(0.022)
Stromal invasion	< 1/2	33	22	11	0.373	12	21	0.817	24	9	0.638
	≥ 1/2	56	31	25	(− 0.111)	18	38	(− 0.043)	37	19	(− 0.069)
Lymphatic metastasis	No	53	33	20	0.660	18	35	1.000	35	18	0.644
	Yes	36	20	16	(− 0.067)	12	24	(− 0.007)	26	10	(0.065)
Parametrium invasion	No	78	48	30	0.341	27	51	0.744	55	23	0.312
	Yes	11	5	6	(− 0.108)	3	8	(− 0.051)	6	5	(− 0.113)
LVSI*	No	15	8	7	0.770	5	10	0.760	10	5	0.760
	Yes	58	35	23	(0.058)	17	41	(− 0.035)	41	17	(0.035)
PNI*	No	42	26	16	0.788	8	34	0.070	30	12	0.774
	Yes	21	12	9	(− 0.046)	9	12	(0.253)	14	7	(− 0.049)
HPV* Infection	Positive	50	32	18	0.145	16	34	1.000	37	1	0.028
	Negative	5	1	4	(0.258)	1	4	(0.075)	13	4	(0.336)
P16	Yes	76	48	28	0.128	24	52	0.349	55	21	0.102
	No	13	5	8	(0.178)	6	7	(− 0.109)	6	7	(0.199)
Ki67	≥ 70%	53	27	26	1.000	11	35	0.048	33	28	0.648
	< 70%	46	19	17	− 0.018	19	24	(− 0.214)	13	15	(0.071)
MMR	dMMR*	17	12	5	0.412	5	12	0.781	12	5	1.000
	pMMR*	72	41	31	(0.109)	25	47	(− 0.041)	49	23	(0.021)
NACT*	Yes	32	18	14	0.659	16	16	0.02	23	9	0.643
	No	57	35	22	(− 0.05)	14	43	(0.258)	38	19	(0.054)
Postoperative therapy	No	5	4	1	0.644	2	3	1.000	4	1	1.000
	Yes	84	49	35	(− 0.102)	28	56	(− 0.032)	57	27	(− 0.06)
Tumor recurrence	No	48	33	15	0.083 (− 0.203)	22	26	0.013 (− 0.278)	11	37	0.071 (− 0.199)
	Yes	41	20	21		8	33		17	24	
Vital status	Alive	59	40	19	0.039 (− 0.236)	23	36	0.162 (− 0.156)	16	12	0.003 (− 0.336)
	Dead	30	13	17		7	1		14	47	

FIGO* International Federation of Gynecology and Obstetrics; LVSI* Lymphovascular invasion; PNI* Perineural Invasion; HPV* Human Papillomavirus; dMMR* deficient mismatch repair; pMMR* proficient mismatch repair; NACT* Neoadjuvant chemotherapy; TPS* the tumor proportion score; ICS* the immune cell score; CPS* Combined Positive Score; r* Spearman’s correlation coefficient;

### Immunohistochemical assessment

**Fig. 1:**
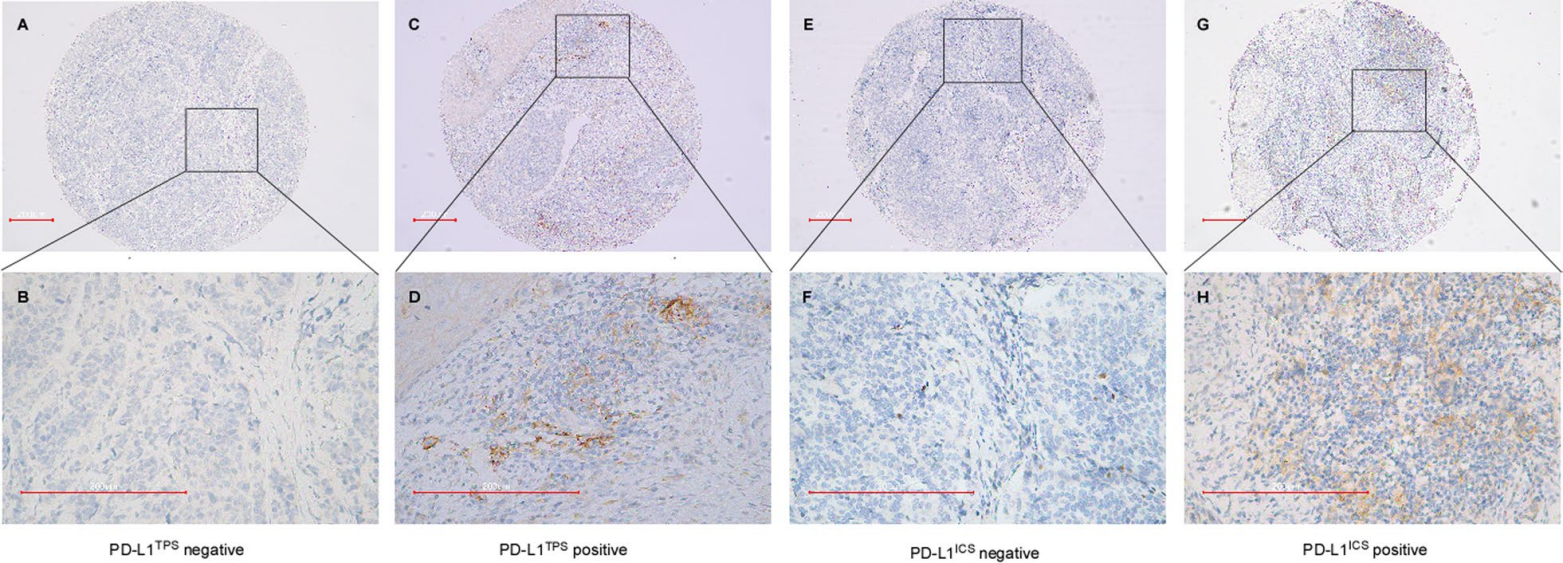
The IHC of PD-L1 in SCNEC patients. **A**, **E** the IHC of PD-L1^TPS^ negative. **B**, **F** The IHC of PD-L1^TPS^ positive. **C**, **G** The IHC of PD-L1^ICS^ negative. **D**, **H** The IHC of PD-L1.^ICS^ positive. (The scale bar is 200 μm)

The immunohistochemical outcomes are summarized in Table 2 and Additional file 1: Fig. S1. PD-L1^CPS^ positivity was seen in 68.5% of SCNEC patients, PD-L1^TPS^ and PD-L1^ICS^ positivity was detected in 59.6% and 33.7% of patients, respectively (Fig. 1). The median PD-L1^CPS^ value was 1 (range = 0–60), while the median PD-L1^TPS^ was 1% (range = 0–15%). The median percentage of PD-L1^ICS^ was 5% (range = 0–60%). There were 19.1% of samples that exhibited dMMR (MLH1/PMS2 loss), 70.6% of which had ≥ 1% PD-L1-positive tumor cells, compared to the proficient mismatch repair (pMMR) group, 56.9% of which showed ≥ 1% PD-L1-positive tumor cells (Spearman’s correlation coefficient(r) = 0.109, *p* = 0.412). The median percentage of Ki-67 index values was 70% (range = 50–95%, interquartile range = 22.5). 87.6% of the SCNEC showed that ≥ 60% of the tumor cells were Ki-67 positive. More than 85.0% of tumor samples showed positive expression for P16 protein. IHC studies revealed that PD1 protein positivity was identified in the immune cells and was found in 50.6% of samples, 10.0% exhibited PD-1 levels above 10%. FOXP3 positive TILs were observed in 62.9% of specimens. The percentage of specimens who had high level of CD3 + , CD20 + , CD4 + , CD8 + , and CD68 + TILs density were 49.4% (median = 6.0%), 56.2% (median = 0.0%), 51.7% (median 3.0%), 50.6% (median 5.0%), and 70.8% (median 2.0%), respectively.

**Table 2:** Correlation Between PD-L1 and TILs of SCNEC

Variable	Total cases		PD-L1^TPS*^		*P*-value (r*)	PD-L1^ICS*^		*P*-value (r*)	PD-L1^CPS*^		*P*-value (r*)
			Positive	Negative		Positive	Negative		Positive	Negative	
CD20	Positive	51	37	14	0.005 (0.307)	25	25	< 0.001 (0.390)	42	8	0.001 (0.377)
	Negative	38	16	22		5	34		19	20	
CD4	High	46	36	10	0.000 (0.394)	18	28	0.370 (0.119)	42	4	< 0.001 (0.507)
	Low	43	17	26		12	31		19	24	
CD8	High	45	36	9	< 0.001 (0.421)	26	19	< 0.001 (0.515)	41	4	< 0.001 (0.492)
	Low	44	17	27		4	40		20	24	
CD68	High	63	42	21	0.056 (0.226)	21	42	1.000 (− 0.12)	49	14	0.006 (0.310)
	Low	26	11	15		9	17		12	14	
CD3	High	45	37	8	< 0.001 (0.467)	23	22	0.001 (0.372)	42	3	< 0.001 (0.540)
	Low	44	16	28		7	37		19	25	
TILs	High	59	44	15	< 0.001	20	39	1.000	48	11	0.001
	Low	30	9	21	(0.429)	10	20	(0.006)	13	17	(0.387)
PD-L1 ^TPS^		53	53	0	–	24	29	0.006 (0.297)	53	0	< 0.001 (0.822)
	Neg	36	0	36		6	30		8	28	
PD-L1 ^ICS^	Positive	30	24	6	0.006	30	0	–	26	4	0.009
	Negative	59	29	30	(0.297)	0	59		35	24	(0.278)
PD-L1 ^CPS^	Positive	61	53	8	< 0.001	26	35	0.009	61	0	–
	Neg	28	0	28	(0.822)	4	24	(0.278)	0	28	
PD-1	Positive	45	33	12	0.01 (0.284)	23	22	0.001 (0.372)	40	5	< 0.001 (0.443)
	Negative	44	20	24		7	37		21	23	
FOXP3	High	56	43	13	< 0.001 (0.457)	25	31	0.005 (0.301)	49	7	< 0.001 (0.532)
	Low	33	10	23		5	28		12	21	

TPS* the tumor proportion score; ICS* the immune cell score l; CPS* Combined Positive Score; r* Spearman’s correlation coefficient

### Correlation of PD-L1 with clinicopathological features

PD-L1^TPS^, PD-L1^ICS^, and PD-L1^CPS^ were associated with many clinicopathological parameters of SCNEC (Table 1). PD-L1^CPS^ positivity was positively associated with HPV infection (r = 0.336, *p* = 0.028). We also showed that PD-L1^CPS^ and PD-L1^TPS^ positivity was associated with a favorable prognosis (r = − 0.336, *p* = 0.003 and r = − 0.236, *p* = 0.039, respectively). Moreover, PD-L1^ICS^ positivity was negatively associated with Ki67 proliferation (r = − 0.214, *p* = 0.048) and tumor recurrence (r = − 0.278, *p* = 0.013).

PD-L1^ICS^ value increased in the patients who received NACT (r = 0.258, *p* = 0.02). However, there was no statistical correlation between PD-L1 levels from TPS, ICS, and CPS levels and MMR status (Additional file 1: Fig. S2), PD-L1^CPS^ levels also had no statistical correlation with P16 expression (Additional file 1: Fig. S3) and the proliferation of Ki67 (Additional file 1: Fig. S3).

### Correlation of PD-L1 with TAICs

**Fig. 2:**
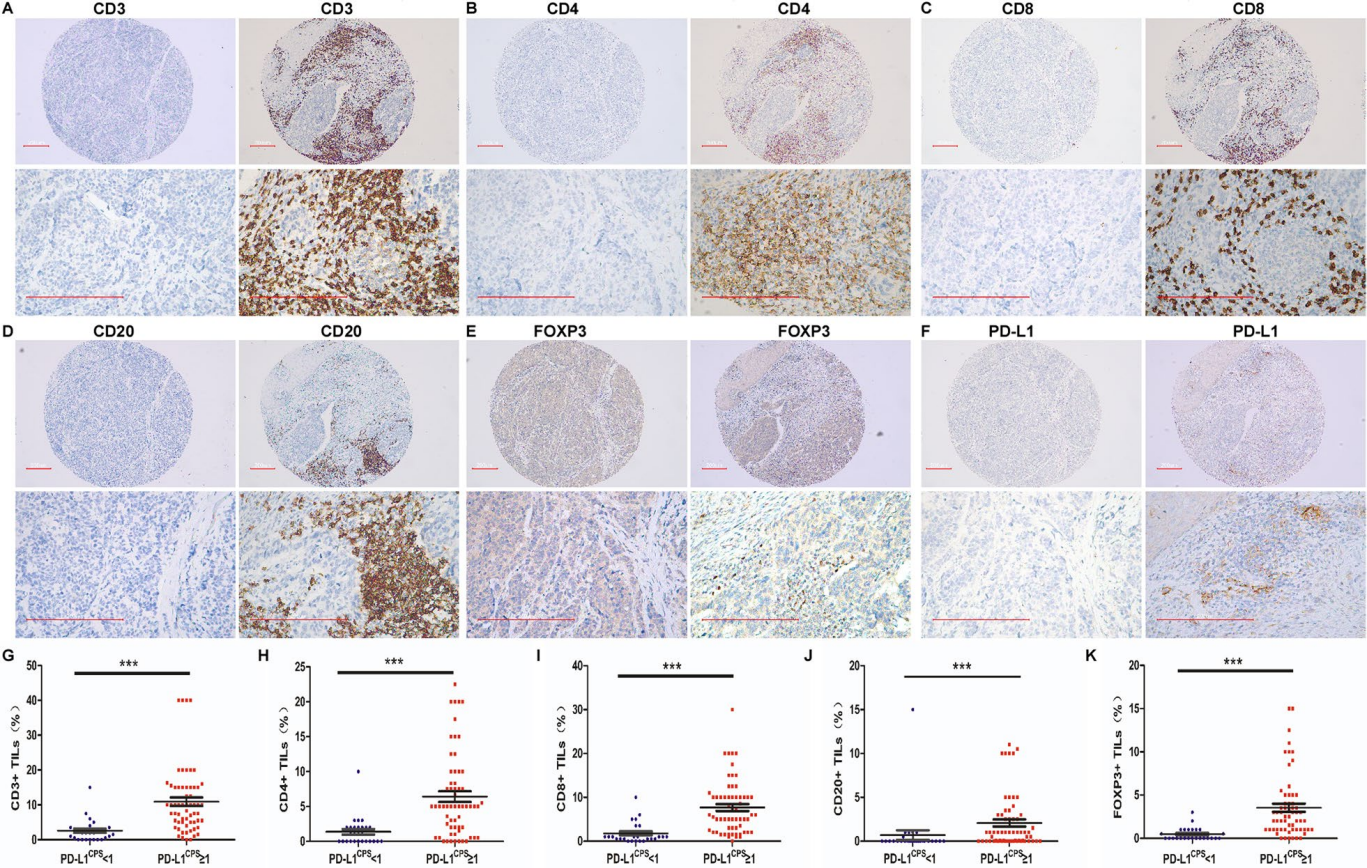
The infiltration by different proportion of TILs in PD-L1^CPS^ negative and PD-L1^CPS^ positive groups. **A**–**E** The IHC of the infiltration by a low (left side) or high (right part) proportion of CD3/CD4/CD8/CD20/FOXP3 positive immune cells in each group. F The IHC of PD-L1^CPS^ negative and PD-L1^CPS^ positive. **G** The infiltration by a higher proportion of CD3 positive immune cells in PD-L1^CPS^ positive groups compared with PD-L1^CPS^ negative groups. **H** The infiltration by a higher proportion of CD4 positive immune cells in PD-L1^CPS^ positive groups compared with PD-L1^CPS^ negative groups. **I** The infiltration by a higher proportion of CD8 positive immune cells in PD-L1^CPS^ positive groups compared with PD-L1^CPS^ negative groups. **G** The infiltration by a higher proportion of CD20 positive immune cells in PD-L1^CPS^ positive groups compared with PD-L1^CPS^ negative groups. **K** The infiltration by a higher proportion of FOXP3 positive immune cells in PD-L1^CPS^ positive groups compared with PD-L1^CPS^ negative groups (The scale bar is 200 μm, *** means *p* < 0.001)

PD-L1 and TAIC levels were found to be significantly positively correlated, especially PL-D1^CPS^ (Table 2; Fig. 2). PD-L1^CPS^ value was positively correlated with PD-1 (r = 0.443, *p* < 0.001), FOXP3 + (r = 0.532, *p* < 0.001), TILs (r = 0.387, *p* = 0.001) and other specific TILs, including CD20 + (r = 0.377, *p* = 0.001), CD4 + (r = 0.507, *p* < 0.001), CD8 + (r = 0.492, *p* < 0.001), CD3 + (r = 0.540, *p* < 0.001), and CD68 + (r = 0.310, *p* = 0.006) immune cells. In addition, PD-L1^TPS^ value was higher in TILs (r = 0.429, *p* < 0.001), such as CD20 + (r = 0.307, *p* = 0.005), CD4 + (r = 0.394, *p* < 0.001), CD8 + (r = 0.421, *p* < 0.001), and CD3 + (r = 0.467, *p* < 0.001) immune cells, and was positively correlated with PD-1 (r = 0.284, *p* = 0.01) and FOXP3 + (r = 0.457, *p* < 0.001) Treg infiltration. Moreover, PD-L1^ICS^ value was also significantly positively correlated with PD-1 (r = 0.372, *p* = 0.001) and FOXP3 + (r = 0.301, *p* = 0.005), as well as CD20 + (r = 0.390, *p* < 0.001), CD3 + (r = 0.372, *p* = 0.001), and CD8 + (r = 0.515, *p* < 0.001) immune cells. Taken together, these results suggest that PD-L1 and immune cell markers such as PD-1, FOXP3 + , CD20 + , CD4 + , CD8 + , CD3 + , and CD68 + interact.

### Prognostic significance of PD-L1

**Fig. 3:**
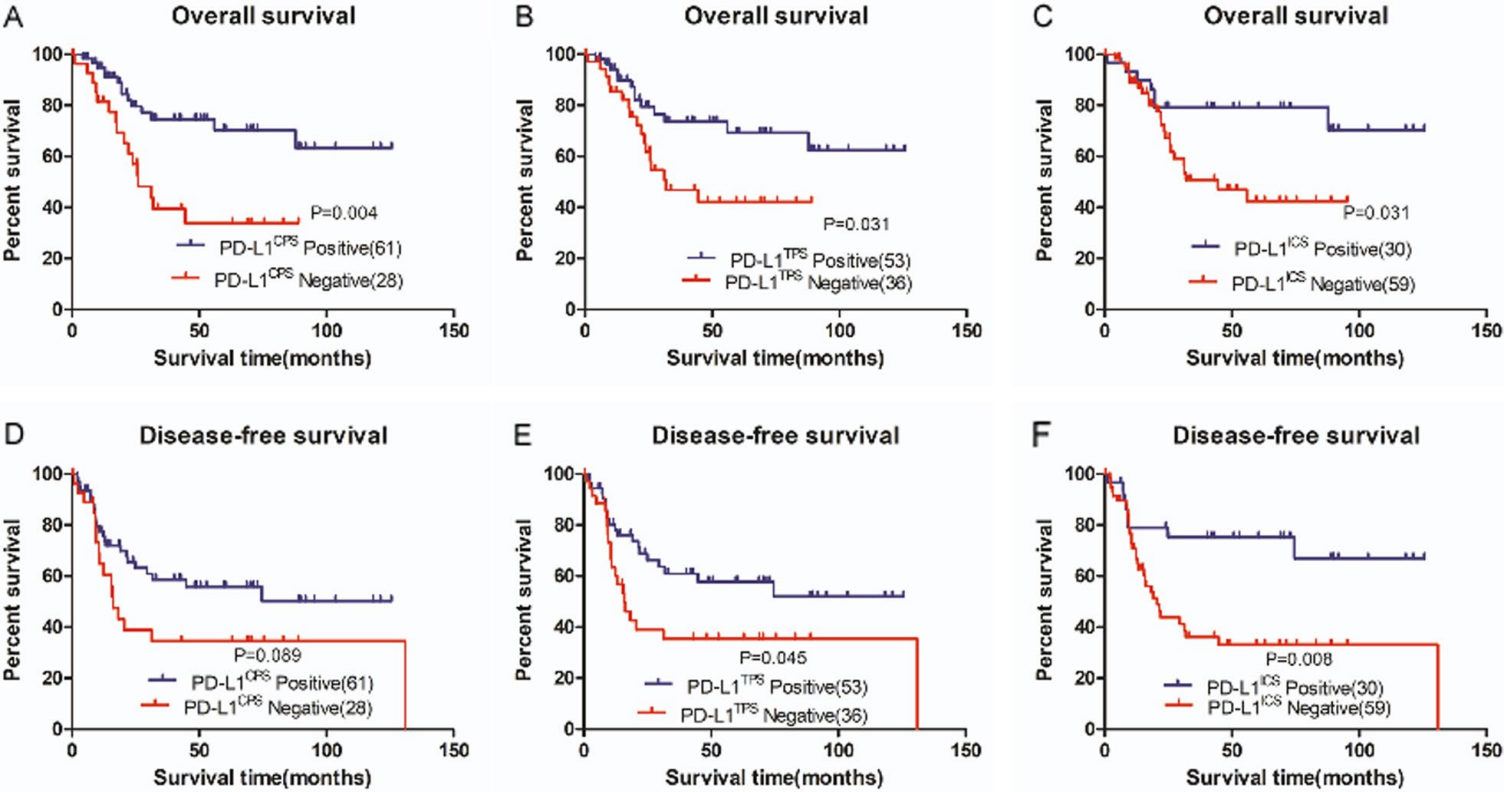
The OS and DFS of PD-L1^CPS^, PD-L1^TPS^ and PD-L1^ICS^ in SCNEC. **A**–**C** The OS of PD-L1^CPS^, PD-L1^TPS^ and PD-L1^ICS^ in SCNEC; **D**–**F** The DFS of PD-L1^CPS^, PD-L1^TPS^ and PD-L1^ICS^ in SCNEC. *P* values are based on the log-rank test

To investigate the prognostic significance of PD-L1, we analyzed overall survival (OS) rates and disease-free survival (DFS) rates among SCNEC patients (Tables 3; 4). Univariate and multivariate analyses indicated that PD-L1^CPS^ and PD-L1^ICS^ positivity were independent prognostic factors that were associated with a favorable survival (HR (95%CI) = 0.363(0.139–0.950), *p* = 0.039 and HR (95% CI) = 0.199(0.050–0.802), *p* = 0.023, respectively) and that PD-L1^ICS^ positivity was an independent indicator of recurrence in SCNEC patients that was associated with a better DFS (HR (95% CI) = 0.124(0.036–0.425), *p* = 0.001). Univariate analysis revealed that PD-L1^TPS^ was associated with a favorable OS and DFS, but there was no statistical significance when assessed with multivariate analyses (Fig. 3). TAIC and MMR status had no statistical impact on survival results.

**Table 3:** Univariate Cox proportional hazards regression analysis for OS and DFS

Variables	OS		DFS	
	HR (95% CI)	*P* value	HR (95% CI)	*P* value
Age (years)(< 45 vs. ≥ 45)	1.035 (0.506–2.119)	0.924	0.993 (0.534–1.847)	0.983
Tumor size(cm)(< 2 vs. ≥ 2, < 4 vs. ≥ 4)	1.658 (0.919–2.993)	0.093	1.254 (0.770–2.042)	0.364
NSE(U/ml) (≤ 15.2 vs. > 15.2)	2.275 (1.046–4.947)	**0.038**	1.037 (0.498–2.161)	0.922
HPV* Infection(Positive vs. Negative)	0.377 (0.109–1.298)	0.122	0.364 (0.107–1.238)	0.106
FIGO* stage (I–II vs. III–IV)	2.033 (0.991–4.170)	**0.048**	1.571 (0.842–2.933)	0.156
Stromal invasion (< 1/2 vs. ≥ 1/2)	1.965 (0.874–4.420)	0.102	2.152 (1.073–4.315)	**0.031**
Lymphatic metastasis (No vs. Yes)	2.033 (0.991–4.170)	0.053	1.571 (0.842–2.933)	0.156
Parametrium invasion (No vs. Yes)	3.251 (1.384–7.633)	**0.004**	2.849 (1.253–6.476)	**0.012**
LVSI* (No vs. Yes vs. Na)	6.489 (0.871–48.312)	0.068	3.750 (1.142–12.311)	**0.029**
PNI* (No vs. Yes vs. Na)	4.479 (1.834–10.938)	**0.001**	3.065 (1.520–6.182)	**0.002**
NACT* (Yes vs. No)	1.716 (0.837–3.521)	0.141	0.998 (0.520–1.916)	0.995
Postoperative therapy (No vs. Yes)	0.749 (0.100–5.620)	0.779	0.292 (0.102–0.831)	**0.021**
P16(No vs. Yes)	0.994 (0.380–2.602)	0.991	1.081 (0.454–2.578)	0.860
Ki67 (≥ 70% vs. < 70%) MMR(dMMR* vs. pMMR*) CD20 (Positive vs. Negative)	0.555 (0.259–1.189)	0.130	0.818 (0.438–1.528)	0.529
	0.687 (0.262–1.797)	0.444	0.580 (0.243–1.384)	0.219
	0.839 (0.408–1.722)	0.631	0.761 (0.410–1.416)	0.389
CD 4 (High vs. Low)	0.761 (0.365–1.588)	0.466	0.997 (0.535–1.857)	0.992
CD 8 (High vs. Low)	0.728 (0.353–1.504)	0.392	0.692 (0.370–1.297)	0.251
CD68 (High vs. Low)	0.928 (0.434–1.986)	0.849	1.146 (0.572–2.294)	0.701
CD3 (High vs. Low)	0.586 (0.278–1.234)	0.160	0.685 (0.366–1.283)	0.238
TILs(High vs. Low)	0.961 (0.460–2.009)	0.916	1.081 (0.556–2.098)	0.819
PD-1(Positive vs. Negative)	0.933 (0.455–1.911)	0.850	1.055 (0.567–1.964)	0.866
FOXP3(High vs. Low)	0.686 (0.332–1.416)	0.308	0.756 (0.403–1.417)	0.383
PD-L1^TPS*^ (Positive vs. Negative)	0.447 (0.215–0.929)	**0.031**	0.529 (0.284–0.987)	**0.045**
PD-L1^ICS*^ (Positive vs. Negative)	0.387 (0.163–0.918)	**0.031**	0.347 (0.158–0.760)	**0.008**
PD-L1^CPS*^ (Positive vs. Negative)	0.345 (0.167–0.711)	**0.004**	0.577 (0.306–1.088)	0.089

FIGO* International Federation of Gynecology and Obstetrics;LVSI* Lymphovascular invasion; PNI* Perineural Invasion; HPV* Human Papillomavirus; dMMR* deficient mismatch repair; pMMR* proficient mismatch repair; NACT* Neoadjuvant chemotherapy; TPS* the tumor proportion score; ICS* the immune cell score; CPS* Combined Positive Score

**Table 4:** Multivariate Cox proportional hazards regression analysis for OS and DFS

Variables	OS^1^		OS^2^		DFS	
	HR (95% CI)	*P* value	HR (95% CI)	*P* value	HR (95% CI)	*P* value
NSE(U/ml) (≤ 15.2 vs. > 15.2)	1.838 (0.591–5-716)	0.293	2.723 (0.683–10.859)	0.156		
FIGO* stage (I-II vs. III- IV)	1.266 (0.418–3.832)	0.677	0.688 (0.188–2.523)	0.573		
PD-L1^CPS*^ (Positive vs. Negative)	0.363 (0.139–0.950)	**0.039**				
Parametrium invasion (No vs.Yes)	1.215 (0.339–4.358)	0.765	1.871 (0.516–6.787)	0.341	2.791 (0.847–9.200)	0.092
PNI* (No vs. Yes vs. Na)	3.617 (1.361–9.616)	**0.010**	5.615 (2.072–15.217)	**0.001**	2.576 (1.135–5.847)	0.024
PD-L1^TPS*^ (Positive vs. Negative)			0.569 (0.210–1.538)	0.266	0.590 (0.266–1.309)	0.194
PD-L1^ICS*^ (Positive vs. Negative)			0.199 (0.050–0.802)	**0.023**	0.124 (0.036–0.425)	**0.001**
Stromal invasion (< 1/2 vs. ≥ 1/2)					2.417 (0.826–7.078)	0.107
LVSI* (No vs. Yes vs. Na)					2.693 (0.638–11.376)	0.178
Postoperative therapy (No vs. Yes)					0.148 (0.040–0.538)	**0.004**

TPS* the tumor proportion score; ICS* the immune cell score; CPS* Combined Positive Score; PNI* Perineural Invasion; LVSI* Lymphovascular invasion

*OS*^*1*^ Multivariate Cox proportional hazards regression analysis of parameters such as NSE, FIGO stage, PD-L1^CPS^, Parametrium invasion and PNI, for overall survival

*OS*^*2*^ Multivariate Cox proportional hazards regression analysis of parameters such as NSE, FIGO stage, PD-L1^TPS^, PD-L1^ICS^, Parametrium invasion and PNI, for overall survival

Considering the clinicopathological characteristics, factors including FIGO stage (HR (95% CI) = 2.033(0.991–4.170), *p* = 0.048), NSE serum level (HR (95% CI) = 2.275(1.046–4.947), *p* = 0.038), parametrium invasion (HR (95% CI) = 3.251(1.384–7.633), *p* = 0.004), and perineural invasion (PNI, HR (95% CI) = 4.479(1.834–10.938), *p* = 0.001) were significantly associated with the survival of SCNEC patients. Moreover, multivariate analysis revealed that PNI was also an independent prognostic factor in SCNEC patients (HR (95% CI) = 3.617(1.361–9.616), *p* = 0.010).

Additionally, there was a negative association between DFS and stromal invasion (HR (95% CI) = 2.152(1.073–4.315), *p* = 0.031), parametrium invasion (HR (95% CI) = 2.849(1.253–6.476), *p* = 0.012), PNI *(*HR (95% CI) = 3.065(1.520–6.182), *p* = 0.002), lymphovascular invasion (LVSI, HR (95% CI) = 3.750(1.142–12.311), *p* = 0.029), and a positive correlation between DFS and postoperative therapy (HR (95% CI) = 0.292(0.102–0.831), *p* = 0.021). Among them, multivariate analysis showed that postoperative therapy (HR (95% CI) = 0.148(0.040–0.538), *p* = 0.004) was an independent recurrence factor in SCNEC patients (Additional file 1: Fig. S4).

## Discussion

The tumor microenvironment, including TILs and tumor associated macrophages (TAMs), has a significant effect on therapy response and clinical results [6, 7, 22, 23]. PD-L1 plays a major role in the induction of tumor cell immune evasion by interacting with its receptor PD-1. Recently, immune checkpoint inhibitor therapy has been at the forefront of anti-tumor treatment showing great efficacy [24]. However, the clinical value of the immune microenvironment in SCNEC tumors has not been sufficiently researched.

This study used the largest cohort of SCNEC cases that has been published to date, investigating the association between PD-L1 and clinicopathological features. PD-L1^CPS^ positivity was seen in 68.5% of SCNEC patients, and PD-L1^TPS^ and PD-L1^ICS^ positivity was detected in 59.6% and 33.7% of patients, respectively. Though without statistical significance, PD-L1 expressed higher in tumor cells in SCNEC patients with dMMR than in those with pMMR. The result was in accordance with a 10-case study in SCNEC patients, which found that PD-L1^TPS^ positivity is seen in 70% of SCNEC and is associated with dMMR [17]. Consequently, dMMR may be able to identify those SCNEC patients who may benefit from PD-L1 inhibitors but needs further investigation.

PD-L1 was found to be significantly positively correlated with levels of TAICs, especially PD-L1^CPS^. PD-L1^CPS^ value was higher in TILs, such as CD3 + , CD4 + , CD8 + , CD20 + , CD68 + immune cells, and was positively correlated with PD-1 and FOXP3 + Treg infiltration with statistical significance. These findings revealed a relationship between the PD-L1/PD-1 pathway and TILs. Much evidence has indicated that the PD-L1/PD-1 pathway is correlated with the maintenance of the immune microenvironment. PD-L1 positivity was correlated with TILs in many tumors, such as osteosarcoma, soft tissue sarcoma, and cervical cancer [17, 25–27]. Several studies showed that PD-L1 has an important effect on sustaining Tregs function in some autoimmune diseases, such as FOXP3 positive TILs and regulating signaling molecules that play a critical role in transforming naive T cells into Tregs [28, 29]. There are two mechanisms for the regulation of PD-L1 TPS including innate and adaptive immune regulation [30]. One is the tumoral constitutive PD-L1 and the other is an adaptive immune response to local inflammatory signals. In our study, PD-L1 may have an effect on the TIL-mediated anti-tumor inflammatory response in SCNEC.

The combined survival rates of SCNEC range from 11 to 54% for all stages [5]. The OS for early-stage SCNEC patients is 30% to 60%, while the 5-year OS for advanced stages is 0% to 17% [31]. Similarly, in our study, the total mortality rate of SCNEC patients was 33.7%, where patient mortality in early stages was 26.4%, which was lower than that of advanced patients (44.4%). Considering the prognosis of SCNEC patients, PNI was an independent prognostic factor and postoperative therapy was an independent recurrence factor in SCNEC patients. PNI may prove to be another metastatic route in cervical cancer [32]. In small cell lung cancer (SCLC), ASCL1 and NEUROD1 could regulate various genes such as SOX2, MYCL1, that contribute to neuronal function and promote perineural invasion [33, 34]. Since PNI is associated with poor survival of SENEC patients, selecting an appropriate surgical method without nerve-sparing methods is significantly important. And postoperative therapy is necessary for some SCNEC patients to reduce the risk of tumor recurrence.

In addition, we identified that PD-L1^CPS^ and PD-L1^ICS^ positivity were independent favorable prognostic indicators and that PD-L1^ICS^ positivity was an independent favorable recurrence factor in SCNEC patients. This study identified a relationship between improved survival of SCNEC patients and PD-L1, indicating that local immunity plays a critical role in limiting tumor progression. Recently, PD-L1 has been classed as an adverse prognostic factor for many types of malignancies [35–38], FOXP3 as the same [27]. One study suggested that PD-L1 is a poor prognostic factor and TILs are favorable prognostic factors in cervical squamous cell carcinoma [39]. The role of PD-L1/PD-1 and TILs in tumors is controversial. A study about SCLC indicated that about 39.3% of patients had PD-L1 protein positivity, and PD-L1 and CD8 + TILs density were associated with better prognoses in patients with SCLC [40].In other studies, PD-L1/PD-1 is also associated with favorable prognoses in various tumors, such as triple negative breast cancer, melanoma, and colorectal cancer [15, 41, 42]. It is difficult to rationalize two diametrically opposed results of PD-L1. With our consideration, one of the reasons that caused this opposed prognostic results of PD-L1 might be related with the different type of cancer and specimens, the different definition of “PD-L1 positive”, and the complex mechanism of PD-L1/PD-1 and TILs.

A next-generation sequencing study of 10 SCNEC patients revealed that genetic alterations involved pathways such as PI3K/AKT/mTOR, MAPK, and TP53/BRCA pathways [43]. In addition, a review of 126 published papers demonstrated that PD-L1, which is regulated by many signaling pathways including PI3K/AKT and MAPK (which can be activated by HPV E6/E7 oncogenes), is associated with HPV-caused cervical cancer carcinogenesis [44]. High-risk HPV infection, especially HPV 18 and HPV 16, participates in the development of SCNEC [45]. In our study, PD-L1^CPS^ positivity was positively correlated with HPV infection, indicating PD-L1 and HPV infection may have some interacting functions which need further research. Another comprehensive sequencing analysis found that PD-L1 and CD8 + TILs are much higher in cervical cancer patients with complete response to chemoradiotherapy than those who did not respond to chemoradiotherapy, indicating an inflammatory tumor microenvironment exists in cervical cancer patients that will respond to chemoradiotherapy [46]. TILs may be a basic predictor of tumor treatment response [47]. All of these studies demonstrate complex mechanisms of PD-L1 and TILs, which may be possible predictors of response to chemoradiotherapy and favorable prognostic indicators in SCNEC patients.

Due to the histopathological similarity to SCLC, the treatment of SCNEC is predominantly based on SCLC, including surgery, radiotherapy, and chemotherapy, which has improved SCNEC patient prognoses to some extent [17]. Researches have suggested that SCLC patients with neuroendocrine (NE)-low subtype are correlated with increased TILs compared to NE-high tumors, providing potential biomarkers for SCLC immunotherapies [48, 49]. Pembrolizumab was approved by the Food and Drug Administration (FDA) for therapy of patients with metastatic or recurrent cervical cancer with PD-L1positivity (as determined by an FDA-approved test in cervical cancer) and patients with metastatic SCLC. More pembrolizumab related clinical research should be carried out in SCNEC.

In conclusion, our study demonstrated that PD-L1 and PD-1 positivity in more than half of SCNEC tumors and may work synergistically with FOXP3 + Treg and other TAICs in support of an adaptive immune response to local inflammatory signals. PD-L1 may be a favorable prognostic factor in SCNEC. Conventional therapies that are in combination with immune modulation treatments, such as a combined strategy to block PD-L1/PD-1 with depletion of FOXP3 + Tregs, may show as a promising treatment for SCNEC patients and may change the course of the disease.

## Supplementary Information

**Additional file 1: Figure S1.** Two SCNEC cases with distinct PD-L1 and immune cell immunohistochemical patterns. Case 1：(A) Hematoxylin and eosin-stained section of SCNEC with the distribution patterns of TILs (B) The IHC of PD-L1^CPS^ negative; (C) The IHC of PD-1 negative; (D) The IHC of infiltration by a low proportion of FOXP3 positive immune cells; (E) The IHC of infiltration by a low proportion of CD3 positive immune cells; (F) The IHC of infiltration by a low proportion of CD4 positive immune cells; (G) The IHC of infiltration by a low proportion of CD8 positive immune cells; (H) The IHC of infiltration by a low proportion of CD20 positive immune cells; (I) The IHC of infiltration by a low proportion of CD68 positive immune cells. Case2：(J) Hematoxylin and eosin-stained section of SCNEC with the distribution patterns of TILs (K)The IHC of PD-L1^CPS^ positive; (L) The IHC of PD-1 positive; (M) The IHC of infiltration by a high proportion of FOXP3 positive immune cells; (N) The IHC of infiltration by a high proportion of CD3 positive immune cells; (O) The IHC of infiltration by a high proportion of CD4 positive immune cells; (P) The IHC of infiltration by a high proportion of CD8 positive immune cells; (Q) The IHC of infiltration by a high proportion of CD20 positive immune cells; (R) The IHC of infiltration by a high proportion of CD68 positive immune cells(The scale bar is 200μm). **Figure S2.** The difference of PD-L1^TPS^, PD-L1^ICS^ and PD-L1^CPS^ in dMMR and pMMR groups. (A) PD-L1 negative in dMMR (MLH1/PMS2 loss) group;(B) PD-L1 positive in pMMR group;(C)The difference of PD-L1^TPS^ in dMMR and pMMR groups(*p*>0.05).(D) The difference of PD-L1^ICS^ in dMMR and pMMR groups (*p*>0.05).(E) The difference of PD-L1^CPS^ in dMMR and pMMR groups. (*p*>0.05). (The scale bar is 200μm). **Figure S3.** The difference of PD-L1^CPS^ in P16 positive and P16 negative groups and Ki67-Low proliferation and Ki67-High proliferation groups. (A) PD-L1^CPS^ positive in P16 negative group (B) PD-L1^CPS^ positive in P16 positive groups; (C) PD-L1^CPS^ negative in Ki67-Low proliferation group; (D) PD-L1^CPS^ positive in Ki67-High proliferation group;(E) PD-L1^CPS^ levels had no statistical correlation with P16 expression (*p*>0.05); (F) PD-L1^CPS^ levels had no statistical correlation with the proliferation of Ki67 (*p*>0.05) (The scale bar is 200μm). **Figure S4.** Kaplan-Meier plots of OS and DFS in SCNEC patients. (A) OS in patients with early-stage and advance-stage.(B)OS in patients with NSE level ≤15.2U/ml and >15.2 U/ml. (C)OS in patients with and without parametrium invasion.(D) OS in patients with and without PNI. (E) DFS in patients with and without PNI. (F)DFS in patients with and without parametrium invasion. (G)DFS in patients with and without LVSI. (H) DFS in patients with stromal invasion level more or less than 1/2. (I)DFS in patients accepting postoperative therapy or not. P values are based on the log-rank test.

## Data Availability

The data that support the findings of this study are available from the RDD website: http://www.researchdata.org.cn/.

## Abbreviations

SCNEC: Small-cell neuroendocrine carcinoma of the cervix
TILs: Tumor-infiltrating immune cells
PD-1: Programmed cell death-1
PD-L1: Programmed cell death protein 1 ligand 1
Tregs: Regulatory T cells
FOXP3 +: Tregs regulated by forkhead box P3 +
dMMR: Deficient mismatch repair
TAIC: Tumor-associated immune cell
FIGO: The International Federation of Gynecology and Obstetrics
HPV: Human papillomavirus
NSE: Neuron-specific enolase
TMA: Tissue microarray
PMS2: PMS1 homolog 2
MSH2: MutS homolog 2
MLH1: MutL homolog 1
MSH6: MutS homolog 6
CD20: Membrane spanning 4-domains A1
Ki-67: Marker of proliferation Ki-67
P16: Cyclin dependent kinase inhibitor 2A
CPS: Combined positive score
TPS: Tumor proportion score
ICS: Immune cell score
SPSS: The Statistical Product and Service Solutions
NACT: Neoadjuvant chemotherapy
EP: The cisplatin and etoposide
DFS: Disease-free survival
OS: Overall survival
TAMs: Tumor associated macrophages
SCLC: Small cell lung cancer
FDA: The Food and Drug Administration
