# Supplementary material for: The prognostic impact of the immune microenvironment in small-cell neuroendocrine carcinoma of the uterine cervix: PD-L1 and immune cell subtypes
Source: Cancer Cell Int. 2022 Nov 14;22:348. doi: 10.1186/s12935-022-02716-6 (PMC9664608; doi:10.1186/s12935-022-02716-6)
Supplement: Supplementary file 1 — Additional file 1: Figure S1. Two SCNEC cases with distinct PD-L1 and immune cell immunohistochemical patterns. Case 1：(A) Hematoxylin and eosin-stained section of SCNEC with the distribution patterns of TILs (B) The IHC of PD-L1CPS negative; (C) The IHC of PD-1 negative; (D) The IHC of infiltration by a low proportion of FOXP3 positive immune cells; (E) The IHC of infiltration by a low proportion of CD3 positive immune cells; (F) The IHC of infiltration by a low proportion of CD4 positive immune cells; (G) The IHC of infiltration by a low proportion of CD8 positive immune cells; (H) The IHC of infiltration by a low proportion of CD20 positive immune cells; (I) The IHC of infiltration by a low proportion of CD68 positive immune cells. Case2：(J) Hematoxylin and eosin-stained section of SCNEC with the distribution patterns of TILs (K)The IHC of PD-L1CPS positive; (L) The IHC of PD-1 positive; (M) The IHC of infiltration by a high proportion of FOXP3 positive immune cells; (N) The IHC of infiltration by a high proportion of CD3 positive immune cells; (O) The IHC of infiltration by a high proportion of CD4 positive immune cells; (P) The IHC of infiltration by a high proportion of CD8 positive immune cells; (Q) The IHC of infiltration by a high proportion of CD20 positive immune cells; (R) The IHC of infiltration by a high proportion of CD68 positive immune cells(The scale bar is 200μm). Figure S2. The difference of PD-L1TPS, PD-L1ICS and PD-L1CPS in dMMR and pMMR groups. (A) PD-L1 negative in dMMR (MLH1/PMS2 loss) group;(B) PD-L1 positive in pMMR group;(C)The difference of PD-L1TPS in dMMR and pMMR groups(p>0.05).(D) The difference of PD-L1ICS in dMMR and pMMR groups (p>0.05).(E) The difference of PD-L1CPS in dMMR and pMMR groups. (p>0.05). (The scale bar is 200μm). Figure S3. The difference of PD-L1CPS in P16 positive and P16 negative groups and Ki67-Low proliferation and Ki67-High proliferation groups. (A) PD-L1CPS positive in P16 negative group (B) PD-L1CPS positive [file 12935_2022_2716_MOESM1_ESM.pdf]

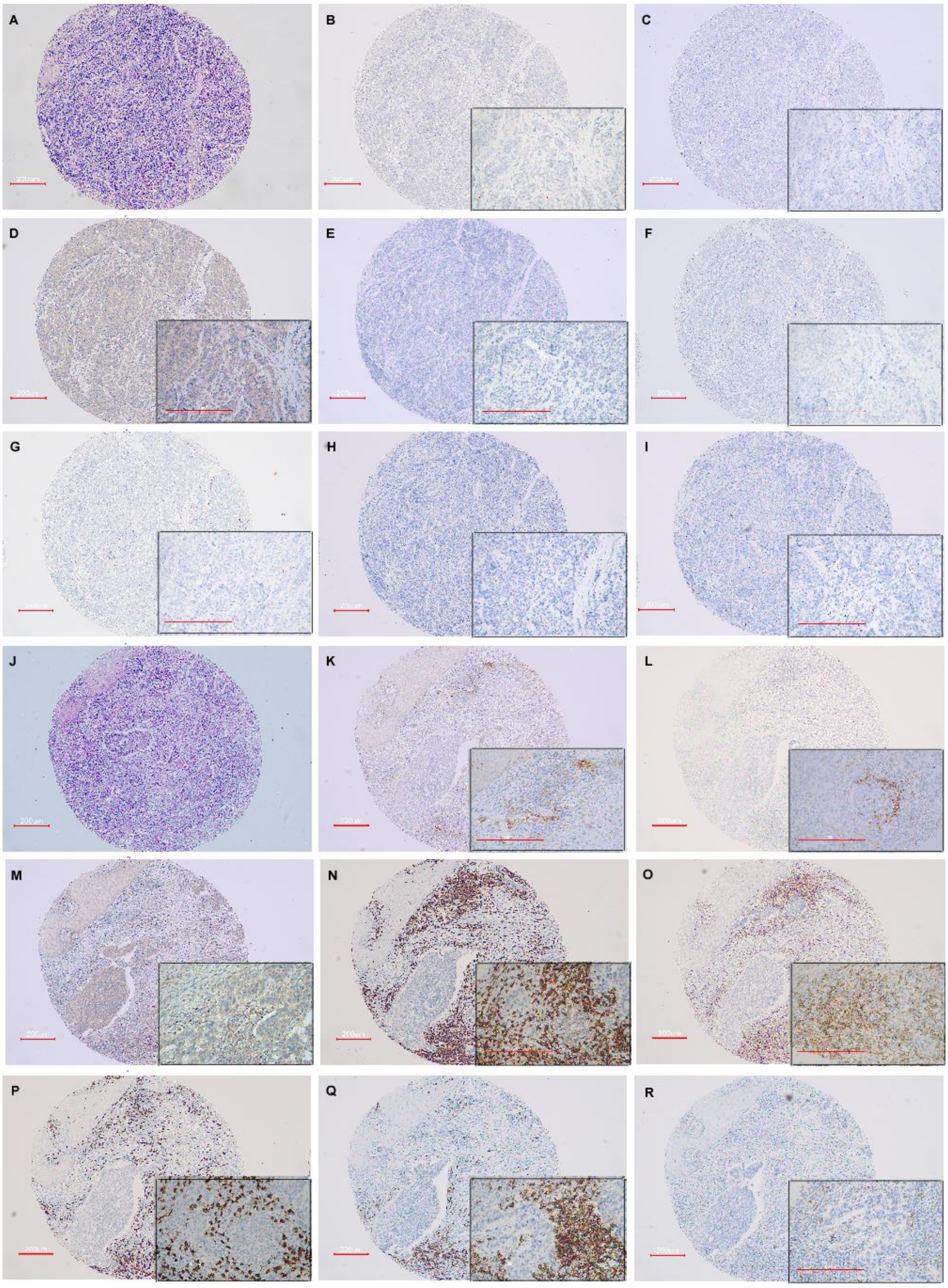

Supplementary Figure 1. Two SCNEC cases with distinct PD-L1 and immune cell immunohistochemical patterns

Case1：(A) Hematoxylin and eosin-stained section of SCNEC with the distribution patterns of TILs (B)

The IHC of PD-L1^{CPS} negative; (C) The IHC of PD-1 negative; (D) The IHC of infiltration by a low proportion of FOXP3 positive immune cells; (E) The IHC of infiltration by a low proportion of CD3 positive immune cells; (F) The IHC of infiltration by a low proportion of CD4 positive immune cells; (G) The IHC of infiltration by a low proportion of CD8 positive immune cells; (H) The IHC of infiltration by a low proportion of CD20 positive immune cells; (I) The IHC of infiltration by a low proportion of CD68 positive immune cells;

Case2：(J) Hematoxylin and eosin-stained section of SCNEC with the distribution patterns of TILs (K)The IHC of PD-L1^{CPS} positive; (L) The IHC of PD-1 positive; (M) The IHC of infiltration by a high proportion of FOXP3 positive immune cells; (N) The IHC of infiltration by a high proportion of CD3 positive immune cells; (O) The IHC of infiltration by a high proportion of CD4 positive immune cells; (P) The IHC of infiltration by a high proportion of CD8 positive immune cells; (Q) The IHC of infiltration by a high proportion of CD20 positive immune cells; (R) The IHC of infiltration by a high proportion of CD68 positive immune cells(The scale bar is 200μm).

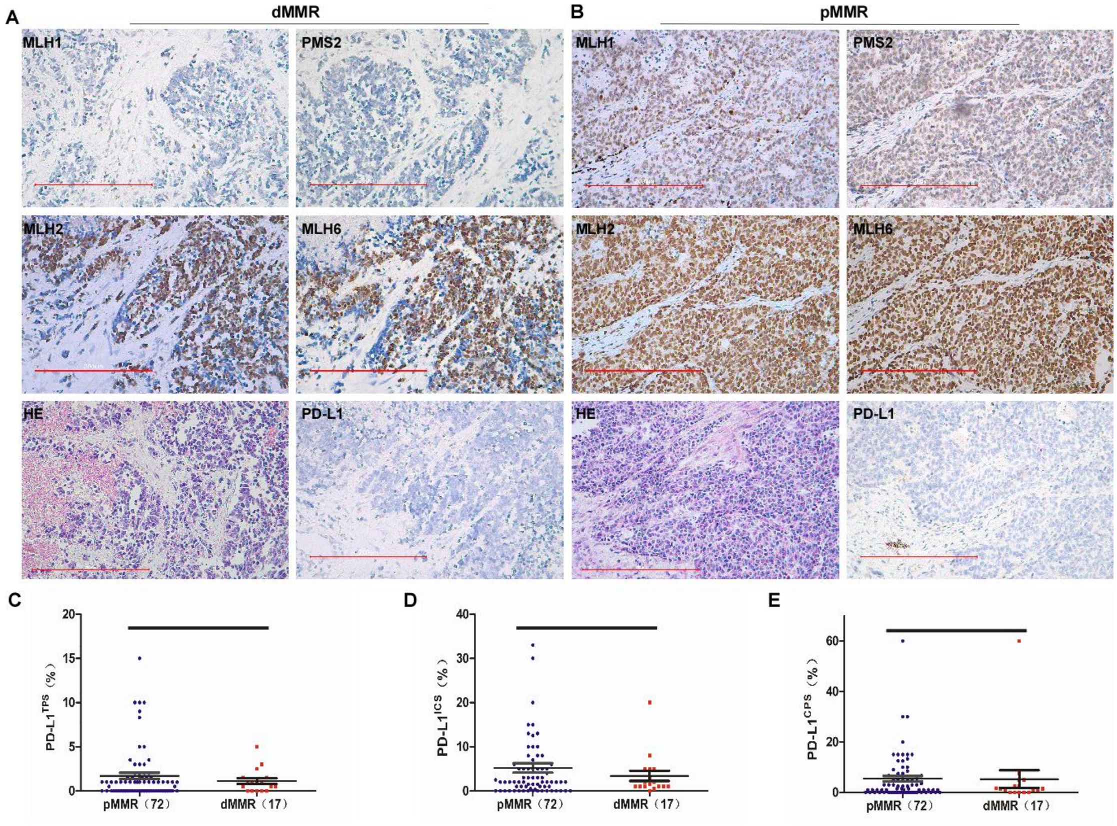

Supplementary Figure 2. The difference of PD-L1^{TPS}, PD-L1^{ICS} and PD-L1^{CPS} in dMMR and pMMR groups.

(A) PD-L1 negative in dMMR (MLH1/PMS2 loss) group;(B) PD-L1 positive in pMMR group;(C)The difference of PD-L1^{TPS} in dMMR and pMMR groups(p>0.05).(D) The difference of PD-L1^{ICS} in dMMR and pMMR groups (p>0.05).(E) The difference of PD-L1^{CPS} in dMMR and pMMR groups (p>0.05). (The scale bar is 200μm)

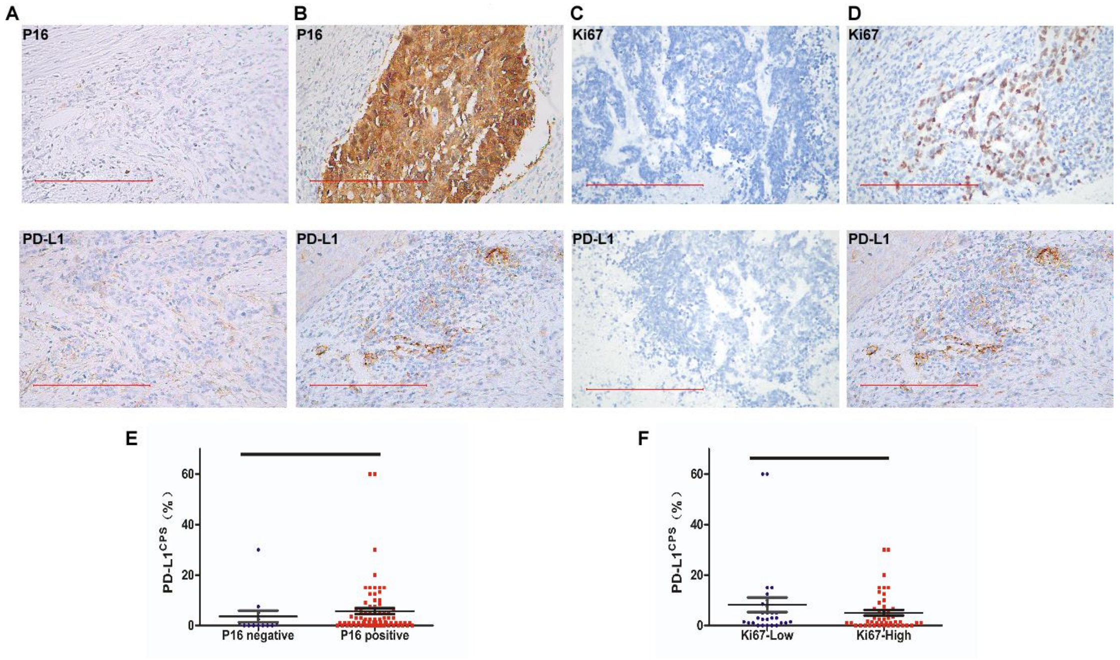

Supplementary Figure 3. The difference of PD-L1^{CPS} in P16 positive and P16 negative groups and Ki67-Low proliferation and Ki67-High proliferation groups.

(A) PD-L1^{CPS} positive in P16 negative group (B) PD-L1^{CPS} positive in P16 positive groups; (C) PD-L1^{CPS} negative in Ki67-Low proliferation group; (D) PD-L1^{CPS} positive in Ki67-High proliferation group;(E) PD-L1^{CPS} levels had no statistical correlation with P16 expression (p>0.05); (F) PD-L1^{CPS} levels had no statistical correlation with the proliferation of Ki67 (p>0.05) (The scale bar is 200μm).

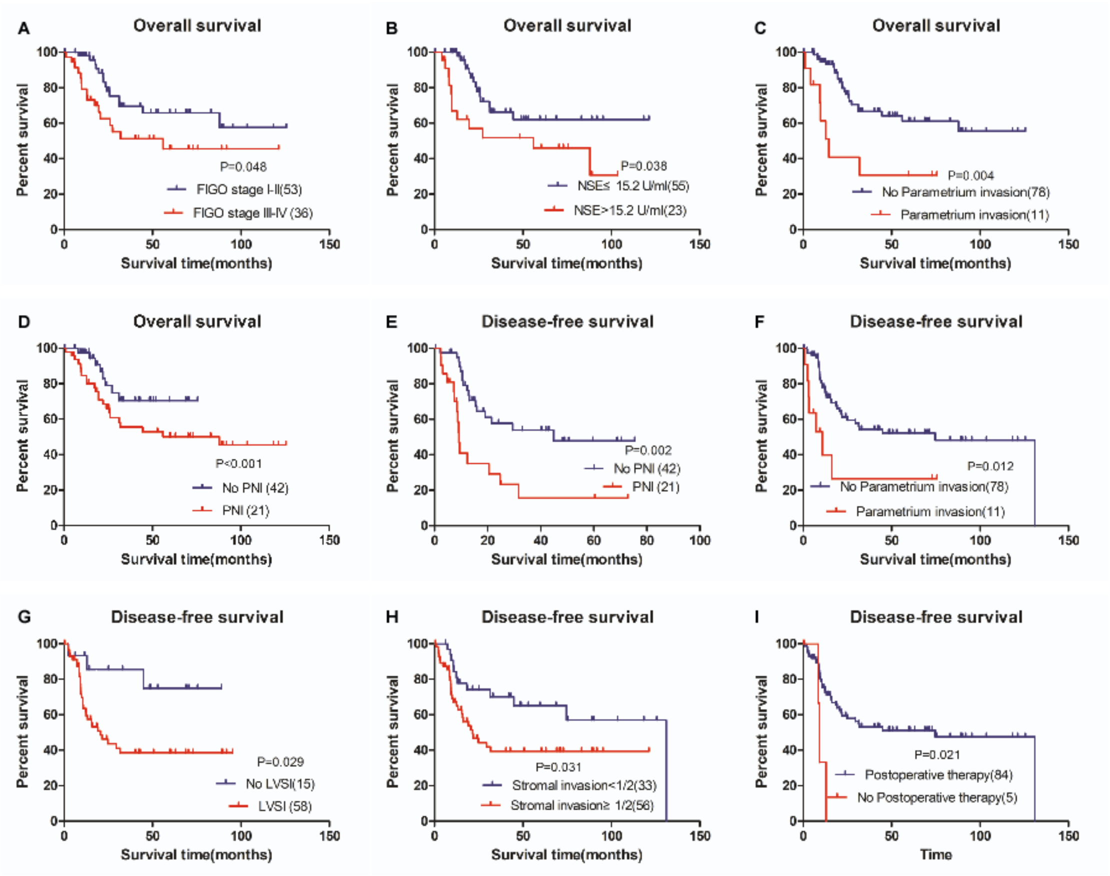

Supplementary Figure 4. Kaplan-Meier plots of OS and DFS in SCNEC patients.

(A)OS in patients with early-stage and advance-stage.(B)OS in patients with NSE level ≤15.2U/ml and >15.2 U/ml. (C)OS in patients with and without parametrium invasion.(D) OS in patients with and without PNI. (E) DFS in patients with and without PNI. (F)DFS in patients with and without parametrium invasion. (G)DFS in patients with and without LVSI. (H) DFS in patients with stromal invasion level more or less than 1/2. (I)DFS in patients accepting postoperative therapy or not. P values are based on the log-rank test.
